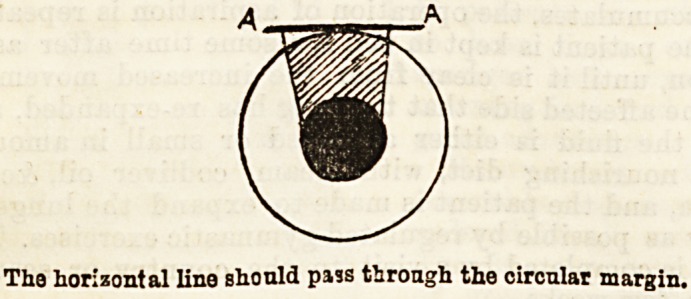# Treatment of Glaucoma

**Published:** 1893-07-29

**Authors:** 


### THE BRISTOL EYE HOSPITAL.

### Treatment of Glaucoma.

The treatment of glaucoma is directed towards relieving the excessive tension of the eyeball, due to the accumulation of the intra-ocular fluids, resulting from an interference with the drainage system, or from excessive secretion of serous fluid during an attack of irido-choroiditis, or from some alteration in the action of the nerves presiding over and regulating the secretion. Some consider the excessive secretion due to sclerotitis, and anatomical changes in the tissues around the canal of Schlemm causing a constriction of the filtration angle. All these causes, whichever of them is correct, point to operation with a view to assist the drainage of the eyeball. Von Graefe introduced the operation of iridectomy as a permanent relief in these cases, and it is, we may say, now universally practised, and the only method of relief that can be perfectly relied on; the conditions observed are to make the iridectomy in the periphery, and to remove a wide portion of the base of the iris.

When a case presents itself without any acute symptoms, beyond tension and occasional blurring or cloudiness of vision in one eye, and pupil slightly dilated, instillations of eserin sulphate dissolved in distilled water, of a strength of ½ grain to 2 grains to the ounce, are prescribed, and this contracting the pupil, will relieve the block in the angle of filtration, and allow the natural drainage of the eyebell to be resumed. But after one or more of these temporary obscurations, or when without them vision is rapidly declining, nothing short of a wide iridectomy is depended on, and the eye is not considered safe until this has been performed.

The operation is performed as follows: The patient having had the eye completely anæsthetised with cocain solution, is placed upon the operating table with the eye to be operated on next to a good light—daylight when possible. The eyelids being separated with a spring speculum, the surgeon takes hold of the conjunctiva near the margin of the cornea at its lowest point, and makes his incision into the anterior chamber at the uppermost margin of the cornea, with a Graefe's triangular knife. Consequently, as the point of the triangular knife enters the corneal-tissue at its uppermost point, the base of the knife must incise the sclerotic as the point passes across the anterior chamber to the pupillary margin. This is best demonstrated by aid of a diagram, showing on an enlarged scale a little triangle of sclerotic on each side of the corneal puncture, for, as we have said before, it is necessary that the iris should be removed at the periphery to establish proper filtration.

The point of the knife having reached the centre of the pupil, it is carefully withdrawn, so that the aqueous escapes slowly. A pair of iris forceps is then introduced, and the margin of the pupil caught by them and withdrawn through the corneal incision. It is drawn first to one angle of the wound, and half divided with scissors, and then to the other angle of the wound, where the other half is similarly treated; this leaves a pupil shaped as below, but as the upper eyelid covers the greater part of it it will not look un-

The horizontal line should pass through the circular margin.

seemly. Eserin is applied, the eyelids closed, and a light pad and bandage carefully put on, so that the edges of the wound are in contact and not overriding, for in that case a little cyst is apt to form, which may cause considerable irritation from rubbing against the eyelid. Another precaution to be observed is to allow the aqueous humour to escape slowly, or else the weakened tissues of the eye may miss the support, and a retinal vessel give way. During the process of healing attention is given to the re formation of the anterior chamber, for until this is accomplished tension may exist. Until this chamber is properly formed and the cicatrix united, the patient is kept at perfect rest in bed and the instillations of eserin continued. It may be necessary to administer hypodermic injections of morphia to obtain this state of rest.

The formation of the cyst above referred to is sometimes put down to the corneal wound healing before the sclerotic incision has united, enabling the aqeous humour still to escape and accumulate under the conjunctiva. Some hold with the view that filtration always goes on through the sclerotic cicatrix, and for that reason sclerotomy has been tried as a permanent cure for intra-ocular tension.

In the unfortunate circumstance of the operation not giving relief it is often necessary to perform an iridectomy downwards, *i.e.*, at the opposite margin of the cornea, or re-incision of the old cicatrix is sometimes practised.

General hygiene and constitutional treatment is also carefully attended to, and the action of the skin and kidneys encouraged. When sclerotomy is performed, a Graefe's cataract knife is used, the pupil is contracted by the use of eserine drops, and the knife is introduced through the sclerotic about 1 mm. from the corneal margin—*i.e.*, a point almost corresponding to that, the one angle of the base of the triangular knife in the former operation incises, and the counterpuncture is made at the point the other angle of the base of the triangular knife would come to, having been passed across the anterior chamber; the sclerotic is then cut by a sawing motion until only a bridge of tissue some 2 mm. in breadth remains undivided, when the knife is carefully withdrawn through the first puncture.

There is always a danger of prolapse of the iris in this operation; if it occurs, attempts are made to return it by aid of a platinum spatula, but should it fail, it is advisable to turn the sclerotomy into an iridectomy. When the eye has been neglected, and is bulging and staphylomatous when the patient presents himself, it is rarely of any use to do anything short of excision of the eyeball.

## Figures and Tables

**Figure f1:**